# Evaluation of endothelial cell-specific molecule-1 as a biomarker of glycocalyx damage in canine myxomatous mitral valve disease

**DOI:** 10.1186/s12917-022-03344-y

**Published:** 2022-07-05

**Authors:** Hee-Jeong Hong, Ye-In Oh, Su-Min Park, Ju-Hyun An, Tae-Hee Kim, Hyung-Kyu Chae, Kyoung-won Seo, Hwa-Young Youn

**Affiliations:** Laboratory of Veterinary Internal Medicine, Department of Veterinary Clinical Science, College of Veterinary Medicine, Seoul National University, Seoul, 00826 Republic of Korea

**Keywords:** Cardiovascular disease, Endothelial glycocalyx, ESM-1, Myxomatous mitral valve disease

## Abstract

**Background:**

Endothelial cell-specific molecule-1 (ESM-1) has emerged as a potential biomarker for cardiovascular disease in humans. Myxomatous mitral valve disease (MMVD) is the most common heart disease in dogs, and we hypothesized that MMVD causes chronic inflammation that increases susceptibility to endothelial glycocalyx (eGCX) damage. In this study, we measured the concentration of ESM-1 in a group of dogs with MMVD and evaluated factors affecting eGCX damage.

**Results:**

Sixty-four dogs (control, *n* = 6; MMVD, *n* = 58) were enrolled in this study. There was no significant difference in serum ESM-1 concentrations among the MMVD stages. The serum ESM-1 concentration was significantly higher in the death group than in the alive group in MMVD dogs. (*p* = 0.006). In five dogs with MMVD, serum ESM-1 concentrations tended to decrease when the cardiac drug (pimobendan, furosemide, and digoxin) dose was increased.

**Conclusions:**

In cases where MMVD progressed to decompensated heart failure with clinical symptoms and resulted in death, the concentration of serum ESM-1 increased significantly. Therefore, ESM-1 could be utilized as a new potential negative prognostic factor in patients with MMVD.

## Background

The glycocalyx is located on the luminal surface of the vascular endothelium, forming a boundary between the blood vessel wall and the bloodstream. The endothelial glycocalyx (eGCX), which consists of membrane-bound negatively charged proteoglycans, glycolipids, glycoproteins, and glycosaminoglycans, has been recognized as a vascular protector [1, 2]. The eGCX participates in the maintenance of vascular tone, prevents the adhesion of leukocytes and platelets to the endothelium, and provides selective permeability and filtration [3]. When the eGCX breaks down for certain reasons (e.g., systemic inflammation), the vascular permeability barrier collapses. Following the collapse, various secondary changes appear, such as the enhancement of the ability of leukocytes to approach the inner wall of the artery, propagation of inflammation, and increasing changes in the endothelial mechano-transduction mechanisms that protect against disease [1, 4, 5]. This allows more fluid to leak from compromised blood vessels, exacerbates interstitial edema, and triggers intravascular coagulation [6–8]. In previous studies, conditions such as septic peritonitis and parvoviral infections have been associated with eGCX damage in dogs [9, 10]. Human studies have shown that conditions such as hypertension, hypercholesterolemia, cardiovascular disease, chronic kidney disease, diabetes, atherosclerosis, hypervolemia (aggressive fluid resuscitation), and ischemia–reperfusion injury can also lead to eGCX degradation [11–19].

Myxomatous mitral valve disease (MMVD) is one of the most common heart diseases [20]. MMVD typically progresses slowly over several years, eventually resulting in chronic heart failure (CHF), pulmonary hypertension (PH), and pulmonary edema. One study found that in dogs with severe decompensated CHF, levels of C-reactive protein, white blood cells, and neutrophil counts were significantly increased compared with compensated heart patients and healthy controls [21]. These results indicate that systemic inflammatory conditions may be associated with severe heart failure and poor prognosis [21]. In humans, a previous study reported a significant correlation between inflammation and the course of heart disease [22]. We hypothesized that canine MMVD patients have chronic inflammation, which accelerates eGCX damage, and that eGCX could be an index of worsening and progression of heart failure.

Currently, there is no gold-standard method for evaluating eGCX. The concentration of serum endothelial cell-specific molecule-1 (ESM-1) can be measured indirectly [23]. ESM-1 is a member of the proteoglycan family that consists of eGCX; therefore, it can be used as an indicator of eGCX damage [24]. In humans, ESM-1 is expressed mainly in the lungs and to a lesser extent in the kidneys and heart muscle cells [25, 26] and the levels of this protein may be elevated by inflammatory conditions or tumor cells [27]. Study on the expression of ESM-1 in tissues have not been conducted in veterinary medicine. Previously, four types of serum eGCX-related biomarkers (ESM-1, syndecan-1, angiopoetin-2, and heparan sulfate) were studied in 10 dogs with parvoviral infection. Among the four indicators, only serum ESM-1 concentratoin was significantly associated with parvoviral infection and a lower survival rate. Based on these results, ESM-1 could be utilized as a prognostic factor for diseases that cause inflammation and eGCX damage, such as parvoviral enteritis [9].

In this study, we evaluated the relationship between eGCX damage and MMVD in dogs, using serum ESM-1 concentrations. The correlation between ESM-1 concentration and other factors such as vital signs, echocardiographic parameters, medication, and underlying diseases was also estimated.

## Results

### Patient data

Sixty-four dogs (control, *n* = 6; MMVD, *n* = 58) were enrolled in this study. MMVD patients were of the following breeds: Maltese (*n* = 20), mixed breed dogs (*n* = 7), Pomeranian (*n* = 6), Poodle (*n* = 6), Shih-Tzu (*n* = 6), Chihuahua (*n* = 4), Spitz (*n* = 3), Yorkshire Terrier (*n* = 2), and one dog of each of the following breeds were included: Bichon Frise, Cocker Spaniel, Coton de Tulear, and Dachshund. In addition, six healthy dogs were included; their breeds were Labrador Retriever (*n* = 3), Golden Retriever (*n* = 2), and German Shepherd (*n* = 1).

Patients without comorbidities accounted for 48% of the MMVD group (*n* = 28). Among comorbid diseases, chronic kidney disease (CKD) was the most common (*n* = 26), followed by chronic pancreatitis (*n* = 9). Other diseases included diabetes mellitus (*n* = 2), protein-losing enteropathy (*n* = 2), idiopathic epilepsy (*n* = 1), Addison’s disease (*n* = 1) and chronic hepatopathy (*n* = 1). These diseases were well controlled in the patient at the time of sampling and were not life-threatening. Eight patients (13.8%) in the MMVD group died during the study. The causes of death were cardiopulmonary edema (CPE; *n* = 5), syncope (*n* = 2), and azotemia (*n* = 1) (Table 1).

**Table 1:** Patient information

**Characteristic**	**Control (*n* = 6)**	**MMVD (*n* = 58)**
**Age (yrs)**	2.667 ± 1.033	12.89 ± 2.378
**Sex, n (%)**		
Male	2 (33%)	4 (7%)
Male castrated	2 (33%)	25 (43%)
Female	0 (0%)	5 (9%)
Female spayed	2 (33%)	24 (41%)
**BCS (9-point scale)**	5.333 ± 0.5164	4.641 ± 1.462
**Weight (kg)**	33.18 ± 8.292	4.265 ± 2.048
**Murmur (6-point scale)**	0	4.125 ± 0.787
**Death, n (%)**	0	8 (13.8%)
**Stage (sampling counts)**		
B1		15 (15)
B2		20 (21)
C		19 (19)
D		4 (9)
**Comorbidity**		
None		28 (48%)
CKD		26 (44%)
Chronic pancreatitis		9 (15%)
Etc		8 (13%)

All data are expressed as mean ± SD value

*Abbreviations*: *BCS* Body condition score, *CKD* Chronic kidney disease, *MMVD* Myxomatous mitral valve disease

The medications included pimobendan (*n* = 50), loop diuretics (furosemide, *n* = 21; torsemide, *n* = 8; furosemide and torsemide, *n* = 2), angiotensin-converting enzyme (ACE) inhibitors (*n* = 15), sildenafil (*n* = 22), spironolactone (*n* = 17), digoxin (*n* = 2), thiazide (*n* = 3), and antibiotics (*n* = 14).

### Correlation between ESM-1 concentration and MMVD stage

**Fig. 1:**
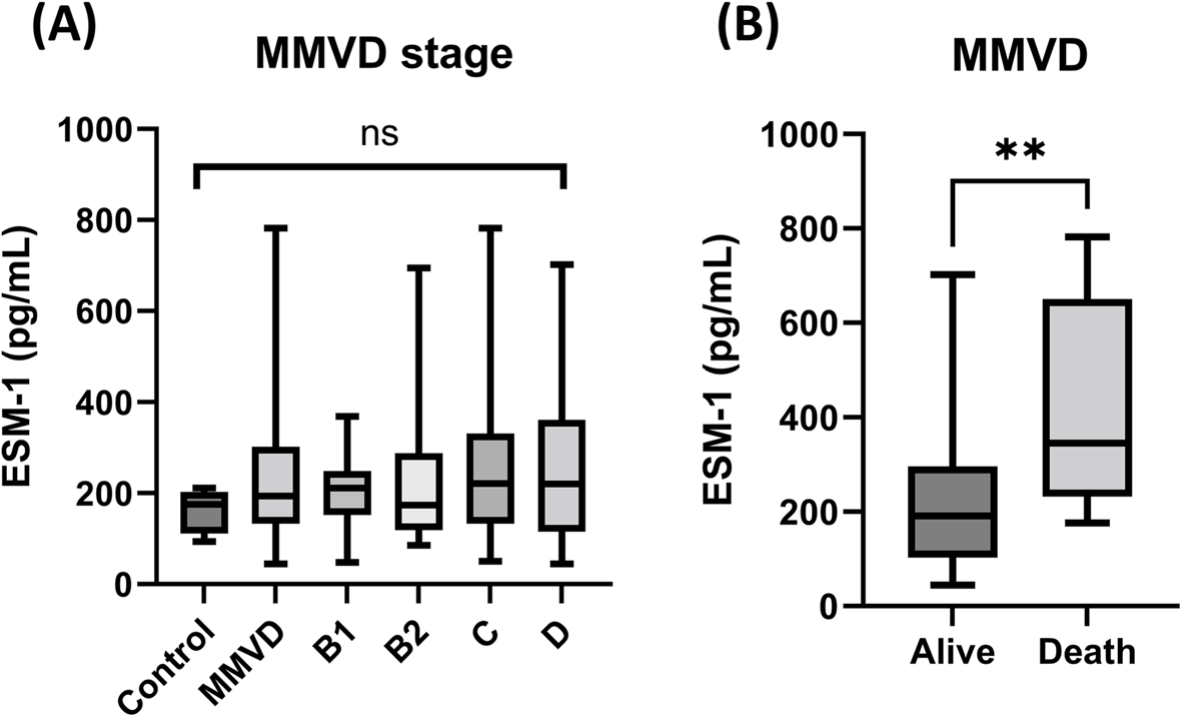
Comparison of serum ESM-1 concentration in control, MMVD, MMVD substages, death, and alive group. **A** No significant difference in serum ESM-1 concentration between the control group and substages of MMVD, **B** ESM-1 concentration was significantly higher in the death group compared to the alive group in MMVD dogs. Abbreviations: ESM-1, endothelial cell-specific molecule-1; MMVD, myxomatous mitral valve disease

**Fig. 2:**
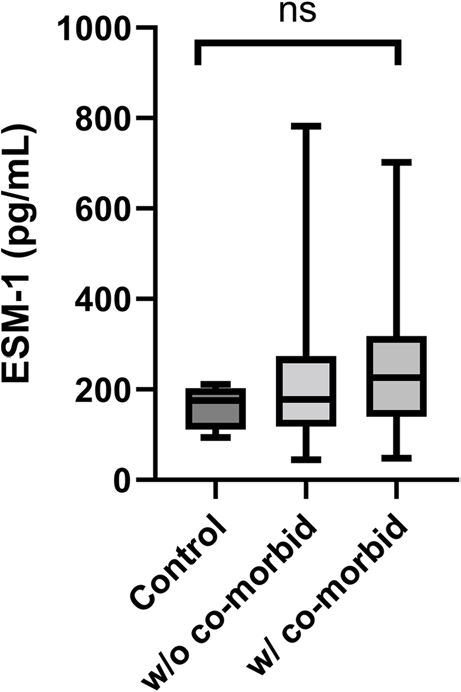
Comparison of serum ESM-1 concentration in control, MMVD group with or without comorbidity. No significant difference was found between patients without comorbidities and patients with comorbidities. Abbreviations: ESM-1, endothelial cell-specific molecule-1; MMVD, myxomatous mitral valve disease

There was no significant difference in serum ESM-1 concentration between the control and MMVD groups and no significant difference between the substages of MMVD (Fig. 1A). Serum ESM-1 concentration was significantly higher in the death group than in the alive group in MMVD dogs (*p* = 0.006) (Fig. 1B). Whether the patient had comorbidities or not did not make a significant difference in serum ESM-1 concentration (Fig. 2). The median values and *p*-values of each comparison of serum ESM-1 concentrations between groups are summarized in Table 2.

**Table 2:** Comparison of the median value of serum ESM-1 concentration

**ESM-1 (pg/mL)**				***P-*value**
**Control**		**MMVD**		0.303^a^
175.2 (111, 202.7)		193.6 (133.2, 301.8)		
**MMVD w/ co-morbid**		**MMVD w/o co-morbid**		0.449^b^
178.0 (117.9, 273.3)		226.6 (139.9, 317.4)		
**Death**		**Alive**		0.006^c^
345.6 (231.8, 650.6)		191. 6 (103.2, 296.6)		
**B1**	**B2**	**C**	**D**	0.886^d^
211.3 (152.4, 249.1)	174.1 (118.9, 287.9)	221.5 (132.8, 331.0)	220.5 (115.1, 361.4)	

Data are presented as median (and IQR). B1, B2, C, D represents the stage of MMVD ACVIM stage

*Abbreviations*: *ESM-1* Endothelial cell-specific molecule-1, *MMVD* Myxomatous mitral valve disease, *IQR* Interquartile range

^a^Comparison between control and MMVD group

^b^Comparison between MMVD w/ co-morbid and w/o co-morbid group

^c^Comparison between death and alive group

^d^Comparison between MMVD stages

### Analysis of other factors influencing ESM-1

To identify the factors affecting serum ESM-1, the *p*-values for age, heart murmur, body condition score, body weight, systolic blood pressure (SBP), radiographic and echocardiographic parameters, and medication dose were analyzed using correlation analysis or Mann–Whitney test (Table 3).

**Table 3:** Predictors for serum ESM-1 concentration

**Factors**	***P*-value**
**Age**	0.954
**BCS**	0.111
**Body weight**	0.228
**Murmur**	0.410
**Systolic BP**^**a**^	**0.009**
**VHS**^**b**^	**0.044**
**VLAS**	0.732
**LA/AO ratio**	0.651
**LVIDdN**	0.516
**Pimobendan**	0.441
**Loop diuretics**	0.072
**ACEi**	0.564
**Spironolactone**	0.523
**Sildenafil**^**b**^	**0.019**
**Antibiotics**	0.367

*Abbreviations*: *ESM-1* Endothelial cell-specific molecule-1, *BCS* Body condition score, *VHS* Vertebral heart score, *VLAS* Vertebral left atrial size, *LA/AO ratio* Left atrium dimension and diameter of the aorta ratio, *LVIDdN* Left ventricular end-diastolic diameter normalized for bodyweight, *ACEi* Angiotensin-converting enzyme inhibitor

^a^Systolic blood pressure had significantly associated with ESM-1 concentration (*p* < 0.01)

^b^VHS and ^b^sildenafil had significantly associated with ESM-1 concentration (*p* < 0.05)

**Fig. 3:**
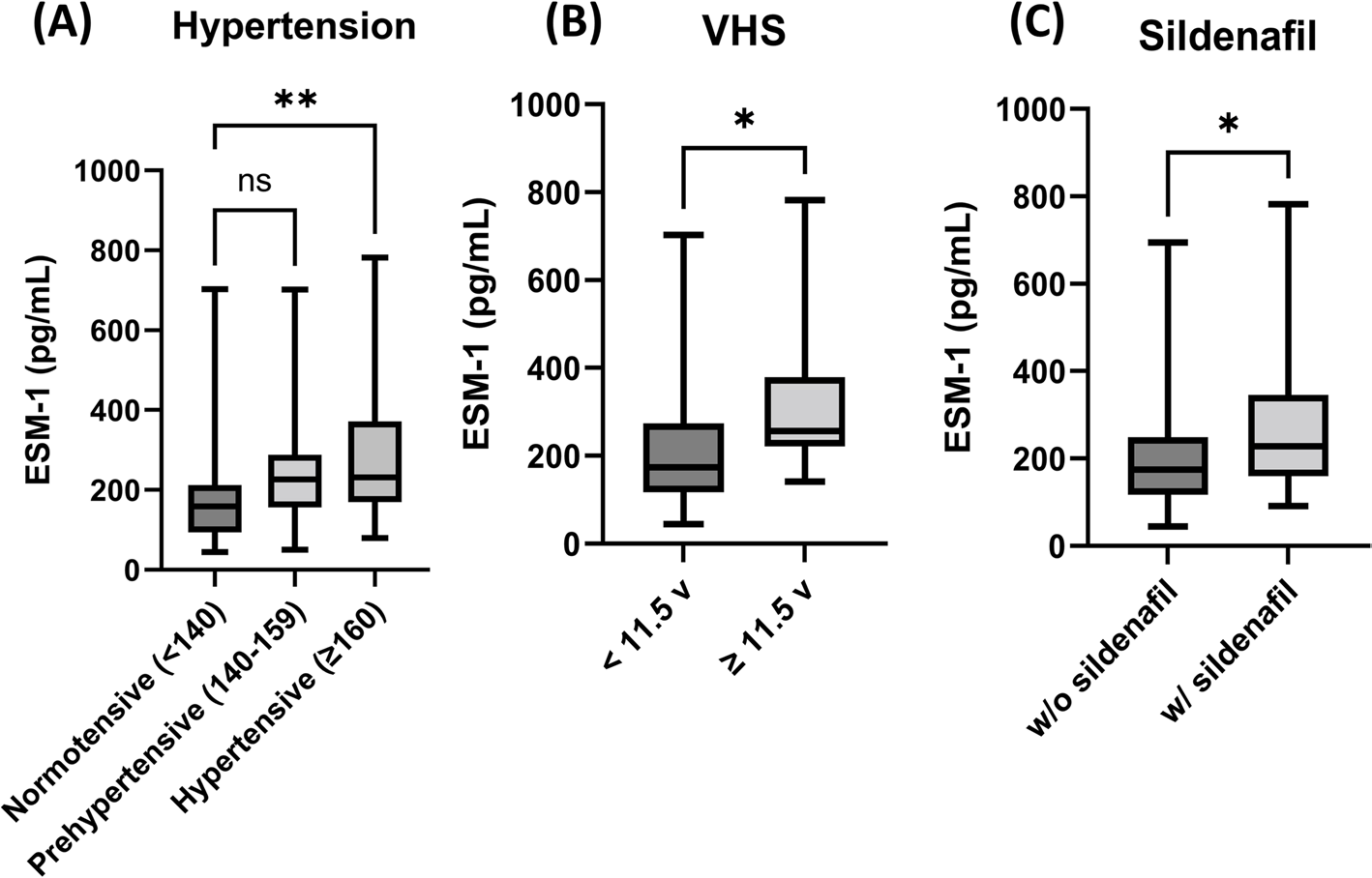
Factors affecting serum ESM-1 concentration. **A** The hypertensive group showed significantly high ESM-1 concentration compared to the normotensive group (*p* < 0.01). Unit of pressure is mmHg in the table. SBP was classified into normotensive (< 140 mmHg), prehypertensive (140 ~ 159 mmHg), and hypertensive (≥ 160 mmHg) according to ACVIM hypertension consensus (2018), **B** When classified as VHS, group above 11.5 v showed significantly increased ESM-1 concentration compared to group under 11.5 v (*p* < 0.05). VHS 11.5, which is considered as general breed cardiomegaly, was used as a cut-off point. **C** Group taking sildenafil showed significantly high ESM-1 concentration compared to a group not taking sildenafil (*p* < 0.05). Abbreviations: ESM-1, endothelial cell-specific molecule-1; VHS, vertebral heart score

In the hypertensive group (SBP ≥ 160 mmHg), the serum ESM-1 concentration was significantly higher than that in the normotensive group (SBP < 140 mmHg) (*p* < 0.01) (Fig. 3A). In the cardiomegaly group (vertebral heart score [VHS] ≥ 11.5 v), the serum ESM -1 concentration was significantly higher than that in the control group (VHS < 11.5) (*p* < 0.05) (Fig. 3B). Finally, when the patients were divided into two groups based on whether sildenafil was taken or not, the group taking sildenafil had a significantly higher ESM-1 concentration than the group that did not take sildenafil (*p* < 0.05) (Fig. 3C).

### Changes in ESM-1 concentrations in response to drug treatment

In 5 of the 58 patients with MMVD, the ESM-1 concentration was evaluated at two-time points. In these patients, the clinical symptoms were not well managed, and the drug dose was increased. We compared the ESM-1 concentration before drug change and at least two weeks after the change.

**Fig. 4:**
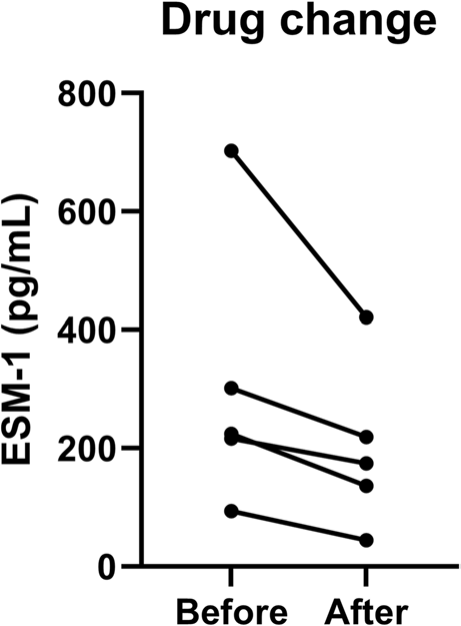
Change in serum ESM-1 concentration before and after the drug change. Serum ESM-1 concentrations decreased and clinical signs resolved in all patients with increased cardiovascular drug dose (*n* = 5) (*p* = 0.0695). Abbreviations: ESM-1, endothelial cell-specific molecule-1

Due to the small study population, the result was not statistically significant, but ESM-1 decreased after the drug dose was increased in all five patients (*p* = 0.0695) (Fig. 4). The median ESM-1 concentration in the pretreatment group was 224.2 pg/mL, whereas, in the post-treatment group, the median ESM-1 was 174.1 pg/mL (Table 4). Overall, case 1 showed a large proportionate decrease (52.7%), and case 2 showed the largest quantitative decrease in ESM-1 (From 702.4 pg/mL to 420.9 pg/mL).

**Table 4:** Serum ESM-1 concentration change and reduction rate before and after drug change

	**ESM-1 (pg/mL)**		Reduction rate (%)
	Before	After	
**Median (IQR)**	224.2 (154.9, 502.1)	174.1 (90.45, 320)	22.3
**Case 1**	93.66357412	44.32312565	52.7
**Case 2**	216.0889026	174.1042404	19.4
**Case 3**	702.4123208	420.973109	40
**Case 4**	301.8416083	218.9565517	27.5
**Case 5**	224.2057542	136.5815375	39.1

*Abbreviations*: *ESM-1* Endothelial cell-specific molecule-1, *IQR* Interquartile range

## Discussion

A recent study has recognized the importance of the eGCX in disease, and an altered eGCX has been associated with increased morbidity and mortality risk in critical illness [28]. ESM-1 has emerged as a potential biomarker in humans, as it is significantly correlated with cardiovascular diseases, including hypertension, coronary artery disease, and heart failure [29–32]. In this study, the difference in serum ESM-1 concentration according to heart disease stage was not significant. We found that serum ESM-1 levels were significantly higher in the death group than the alive group (Fig. 1B). Serum ESM-1 concentration in the death group was measured within 3 weeks before death, and the cause of their death is presumed to be cardiovascular or cardiorenal syndrome. We also attempted to determine whether changes in the eGCX were aggravated by comorbidities. However, there was no significant difference in serum ESM-1 concentration between the groups with and without comorbidities (e.g., CKD, chronic pancreatitis, etc.) (Fig. 2).

Based on these results, it is considered that although MMVD progresses through different stages, the concentration of ESM-1 does not increase if the disease is well managed. Even in the group of dogs with MMVD ACVIM stage C to D, most patients were well managed at the time of blood sampling, and thus a significant increase in eGCX biomarker may not have occurred. However, when MMVD progressed to decompensated heart failure and resulted in death, serum ESM-1 concentration increased significantly. A previous human study reported that serum syndecan (an eGCX biomarker) concentrations in a group of patients with acute decompensated heart failure were higher than those in patients with CHF and healthy controls, and were associated with a decreased survival rate [33]. Based on this study, ESM-1 has the potential to be a negative prognostic factor in dogs.

The factors affecting ESM-1 were analyzed in all dogs with MMVD. First, ESM-1 expression was significantly higher in the hypertensive dogs than in normotensive dogs (Fig. 3A). Hypertension is a well-known factor that damages the eGCX in humans, as increased blood pressure is associated with vascular stretch and reduction in NO bioavailability [32, 34]. In hypertensive human patients, ESM-1 concentration correlates with the presence and severity of coronary artery disease, and the clinical benefits of ACE inhibitors include lower blood pressure and prevention of cardiovascular disease [35]. Considering that ESM-1 is elevated in hypertensive dogs, hypertension is considered to affect eGCX, and its possible effects on cardiovascular disease in dogs remain to be elucidated.

Dogs treated with sildenafil had a significantly higher ESM-1 concentration than the dogs that were not treated with sildenafil (Fig. 3C). As most dogs with PH are already taking sildenafil, there may be a correlation between PH and ESM-1, but the effect of sildenafil on ESM-1 could not be excluded. In humans, PH pathogenesis is associated with endothelial dysfunction [36, 37]. A rat model of PH showed significantly higher concentrations of eGCX shedding ingredients, including heparin sulfate proteoglycan, hyaluronan acid, and syndecan-1, than controls, suggesting that disruption of the glycocalyx is involved in the pathogenesis of PH [38]. Therefore, the development of PH seems to be associated with eGCX damage in dogs, but further studies in dogs with PH before starting sildenafil are needed to clarify this correlation.

The study population was relatively small, and thus a larger sample size is required to confirm the findings. As control group, young and healthy large breed dogs were selected, so there is a limitation that they do not match the age and breed of diseased dogs. The difference in the expression level of serum ESM-1 by breed and age has not been revealed so far, so additional research is needed. In addition, dogs were taking various cardiac drugs, and the effects of these drugs on ESM-1 could not be confidently excluded. Monitoring the change in ESM-1 concentration according to the progression of heart disease in multiple patients over a long period will clarify the correlation between various variables. Lastly, although we hypothesized that inflammatory changes caused by canine heart disease may increase the serum concentration of ESM-1, serum levels of CRP were not measured in every dogs and not correlated with serum ESM-1, remaining as a limiting point. Despite these limitations, in this study, ESM-1 concentrations were significantly increased in patients with poor prognosis.

## Conclusion

ESM-1 may be used as a new potential tool to predict mortality in patients with MMVD. A high ESM-1 level does not always act as a negative prognostic factor; however, considering that the ESM-1 level was significantly increased in the death group compared with the MMVD group, examining the ESM-1 level would be helpful in the identification of patients who would benefit from intensive medical treatment. The level of ESM-1 decreased with the improvement in clinical symptoms after increasing the dose of cardiovascular drugs. It could be useful to continuously evaluate ESM-1 in one patient and to assess the impact of therapeutics.

## Materials and methods

### Case selection

This study was conducted at the Veterinary Medical Teaching Hospital of the College of Veterinary Medicine, Seoul National University.

Sixty-four client-owned dogs of various breeds were recruited, and information on weight, sex, and age was collected. Fifty-eight dogs with MMVD (weight, 1.19 ~ 10 kg; age, 4–17 years) and six healthy dogs (weight, 25 ~ 43.4 kg; age, 2–4 years) who visited the hospital between October 2021 and February 2022 were included in the study. The MMVD groups were divided into four groups (B1, B2, C, and D) according to the American College of Veterinary Internal Medicine (ACVIM) consensus classification [39]. The MMVD staging system describes 4 stages as follows. Stage A dogs are at high risk for developing heart disease but do not currently have any structural abnormality. Stage B describes asymptomatic dogs with structural abnormality. Stage B1 refers to dogs without significant evidence of cardiac remodeling while stage B2 denotes dogs that meet radiographic and echocardiographic criteria of cardiac remodeling and are expected to benefit from pharmacological treatment. Stage C describes dogs with current or past clinical signs of CHF secondary to MMVD. Stage D refers to dogs with end-stage MMVD, with signs of CHF refractory to standard treatment. All dogs underwent a general physical examination, cardiac/pulmonary auscultation, thoracic radiography, and echocardiography. As a control group, healthy dogs that visited the hospital for a health checkup or blood donation with no specific blood test abnormalities and no murmur on auscultation were selected for this study.

Animals that were not well managed or had other medical conditions that could significantly affect survival were excluded. Dogs with respiratory problems and cancers at any sites excluded from this study. Dogs with congenital heart disease (ex. Patent ductus arteriosus, septal defect), dilated cardiomyopathy, or heartworm infection were also excluded because these conditions could affect the patient’s mitral valve dysfunction.

### Data collection

The medical records of the patients were retrieved on the day of blood collection and the following information was collected: signalment, vital signs, thoracic radiography, echocardiography, comorbidity, and medication history. Thoracic radiographic parameters (VHS and vertebral left atrial size), echocardiographic parameters (left atrial-to-aortic diameter ratio, percentage of fractional shortening, left ventricular end-diastolic diameter normalized for body weight, E wave velocity of the mitral valve inflow, and tricuspid regurgitation pressure gradient) were included. Medication history (pimobendan, loop diuretics [furosemide, torsemide], spironolactone, ACE inhibitor, sildenafil, thiazide, digoxin, and antibiotics) was compiled.

### Re-evaluation of serum ESM-1 in the same patient

In the same patient, if there was a drug change or heart disease progression to the next stage, the sampling was performed once more. In five dogs, serum ESM-1 concentration was re-evaluated (Table 4). Cases 1, 2, and 3 were in stage D and showed early signs of CPE on radiography and an increase in sleeping respiratory rate. Pimobendan 0.3 mg/kg/day and furosemide 2 mg/kg/day were prescribed to case 1, pimobendan 0.3 mg/kg/day to case 2, and digoxin 0.005 mg/kg/day to case 3. In case 4, the patient exhibited exercise intolerance and frequent coughing. MMVD progressed from stage B1 to stage B2, and treatment with pimobendan 0.5 mg/kg/day was started. Case 5 repeatedly had ascites due to right-sided heart failure, and the patient was prescribed pimobendan 0.2 mg/kg and furosemide 2 mg/kg.

### Sample collection

Blood samples (2 mL) were collected by venipuncture of the jugular or cephalic vein. The collected blood was placed into tubes without anticoagulant, centrifuged at 2000 × g for 5 min at 4 °C, and the serum samples were extracted. The samples were stored at -80 °C and defrosted before enzyme-linked immunosorbent assay (ELISA) analysis.

### Biomarker measurements (endothelial glycocalyx related biomarkers)

Serum ESM-1 levels were measured using a canine ESM-1 commercial ELISA kit (MyBioSource, USA), according to the manufacturer’s protocol. All samples were run in duplicate, and the mean value of the wells was used. The intra-assay coefficients, inter-assay coefficients, and minimum detectable concentrations were < 10%, < 10% and 1.0 pg/mL.

### Statistical analysis

Statistical analyses were performed using commercially available statistical software (Prism 9.3.1). Descriptive statistics for continuous variables are presented as median (interquartile range). Normality was checked using the Shapiro–Wilk test. Differences among groups were evaluated using the Mann–Whitney test or the Kruskal–Wallis test. A correlation analysis model was used to estimate the correlation between ESM-1 concentration and multiple variables obtained from the medical records. In all comparisons, a probability value of *p* < 0.05 was considered statistically significant, unless otherwise stated.

## Data Availability

All data generated or analyzed during this study are included in this study, but any additional information can be provided by the corresponding author upon reasonable request.

## Abbreviations

ACE: Angiotensin-converting enzyme
ACVIM: American College of Veterinary Internal Medicine
CHF: Chronic heart failure
CKD: Chronic kidney disease
CPE: Cardiopulmonary edema
eCGX: Endothelial glycocalyx
ELISA: Enzyme-linked immunosorbent assay
ESM-1: Endothelial cell-specific molecule-1
MMVD: Myxomatous mitral valve disease
PH: Pulmonary hypertension
SBP: Systolic blood pressure
VHS: Vertebral heart score
